# “Population structure of drug-susceptible,—resistant and ESBL-producing *Escherichia coli* from community-acquired urinary tract”

**DOI:** 10.1186/s12866-016-0681-z

**Published:** 2016-04-11

**Authors:** Frederik Boëtius Hertz, Jesper Boye Nielsen, Kristian Schønning, Pia Littauer, Jenny Dahl Knudsen, Anders Løbner-Olesen, Niels Frimodt-Møller

**Affiliations:** Department of Clinical Microbiology, Hvidovre University Hospital, Copenhagen, Denmark; Department of Biology, University of Copenhagen, Copenhagen, Denmark; Depatment of Clinical Microbiology, Rigshospitalet, Copenhagen, Denmark

**Keywords:** *Escherichia coli*, Lineages, Urinary tract infection, Community-acquired, ESBL

## Abstract

**Background:**

*Escherichia coli* is the most common cause of urinary tract infection (UTI). The pathogenic isolates are becoming increasingly resistant to antibiotics; with a worldwide dissemination of resistant sequence types (ST). We characterized three different uropathogenic *E. coli* populations, from non-hospitalized patients to describe the genetic kinship between resistant and susceptible isolates. We studied the populations by use of multi-locus sequence typing (MLST) and abbreviated-multi locus variable number of tandem repeat analysis (a-MLVA). Urine samples submitted for testing, by general practitioners, were identified at Dept. of Clinical Microbiology at Hvidovre Hospital, Denmark, from Oct. 2011 to July 2012. We included 94 fully susceptible*,* 94 resistant (non-ESBL) and 98 Extended Spectrum Beta-lactamases- (ESBL)-producing *E. coli* isolates.

**Results:**

The ESBL population was dominated vastly by ST131 (51 %), ST38 (9 %) and ST69 (6 %). In the resistant group ST69 (18 %), ST73 (11 %) and ST131 (15 %) were the largest clusters. In the susceptible population more STs and a-MLVA codes were identified compared to the other groups and ST73 and ST95 were found as the only clusters with 16 % and 6 %, respectively. Ninety-eight per cent of the ESBL-producing *E. coli* isolates were CTX-M-producers.

**Conclusion:**

ST131 dominated the population of community-associated uropathogenic ESBL-producing *E. coli,* but was less frequent among non-ESBL-producing *E. coli.* The fully susceptible *E. coli* population was a much more diverse group than the resistant and ESBL-producing *E. coli* populations. Overall, these findings suggest that dominant ESBL-producing lineages are derived from UPEC lineages already established in the general UPEC population.

**Electronic supplementary material:**

The online version of this article (doi:10.1186/s12866-016-0681-z) contains supplementary material, which is available to authorized users.

## Background

*Escherichia coli* is the most common Gram-negative extraintestinal pathogen and the primary cause of urinary tract infection (UTI) [1, 2]. It is a highly heterogonous species with certain lineages becoming increasingly resistant to antibiotics [2–5]. Thus, resistant *E. coli* isolates are emerging worldwide and especially *E. coli* producing extended-spectrum beta-lactamases (ESBLs) have been reported numerous times around the world [2, 6]. Now *E. coli* isolates primarily produce ESBLs belonging to the enzyme family CTX-M [2, 6]. The dissemination of ESBL-producing *E. coli* in hospital settings as well as in the community has been reported as spread of extraintestinal pathogenic *E. coli* (ExPEC) belonging to a limited number of lineages or sequence types (STs) [2, 6]. However, only a few studies have specifically studied the heterogeneity among susceptible, resistant and ESBL-producing *E. coli* [2–4, 6]. Interestingly, dominating ExPEC lineages, like ST73 and ST95 continue to be common causes of UTI, but are rarely multidrug resistant and seldom associated with ESBL production*.* These lineages have previously been sub-typed by different typing methods showing the existence of several subclones. An abbreviated-multi locus variable number of tandem repeat analysis (a-MLVA) method has previously been evaluated and proved efficient in the characterization of local ESBL-producing *E. coli* in hospital settings where the method typed congruent with multi-locus sequence typing (MLST) [7]. However, MLST, in spite of its clear advantage of having an internationally nomenclature, is laborious and needs sequencing where MLVA can be used as a fast and low cost method for screening larger population.

Therefore, we here present a descriptive investigation of three *E. coli* populations all from non-hospitalized patients. The characterization is performed by use of a-MLVA and subsequent MLST of identified a-MLVA codes.

## Methods

### Research ethical approvals

Approvals for this study were granted by the Danish Health and Medicines Authority, Statistics Denmark (DST) and The Regional Committee of Danish Data Protection Agency. Patient notification was declared not necessary by the Regional Committee of Health Research Ethics Committee (HVH-2012-017, J.nr. 3-3013-230/1/KWH and H-4-2012-088).

### Strain collections

The Department of Clinical Microbiology at Hvidovre Hospital, Denmark (DCM) provides services to more than 450 general practitioners in an area of approximately 950.000 inhabitants. From the 1^st^ of October 2011 to 30^th^ of June 2012 we collected 286 *E. coli* isolates from patient urine samples, submitted from general practices. Hence, from non-hospitalized patients, one unique strain was collected per patient and isolates were divided into three susceptibility groups: (i) ESBL-producing *E. coli,* (ii) *E. coli* resistant to at least one of 17 tested antibiotic (ampicillin, cefuroxime, aztreonam, ampicillin/clavulanic acid, piperacillin/tazobactam, mecillinam, ceftazidime, cefpodoxime, meropenem, ciprofloxacin, sulfamethoxazole, trimethoprim, tetracycline, gentamicin, tobramycin, nitrofurantoin, fosfomycin) but without an ESBL phenotype and (iii) fully susceptible *E. coli.* Thus, in this study we included 98 ESBL-producing *E. coli* isolates, 94 resistant isolates (non-ESBL) and 94 fully susceptible *E. coli.*

### Strain identification and susceptibility testing

The UPEC isolates were all identified at species level by MALDI-TOF MS (Bruker, Germany) and antimicrobial susceptibility testing was performed by Disk Diffusion Test Methodology as described in the European Committee on Antimicrobial Susceptibility Testing (EUCAST, Version 1.0, December 18, 2009) or by The Vitek 2 automated system by use of cards AST-N209 and AST-N122 (bioMérieux, France). Methods were performed according to guidelines of DCM in agreement with direction from manufacturers and as described elsewhere [8–11]. The ATCC 25922 *E. coli* was used for quality control and susceptibility interpreted as recommended by EUCAST (www.eucast.org/clinical_breakpoints/). Resistance to cefpodoxime was used as an indicator for ESBL-production. Isolates resistant to cefpodoxime therefore had potential ESBL phenotypes identified by a double-disk diffusion method; here performed as combined-disk diffusion using a AmpC + ESBL detection set (MAST®, Merseyside, UK) as previously described [10].

### Molecular characterization

#### ESBL-genotyping

ESBL-producing *E. coli,* phenotypically recognized by the double-disk diffusion MAST® test, were screened for the presence of *bla*CTX-M genes by multiplex PCR assay, detecting alleles encoding the five CTX-M groups 1, 2, 8, 9 and 25 as previously described [7, 12]. Positive samples were re-amplified and products were sequenced to identify the exact genotypes of CTX-M [7, 12].

#### MLVA and MLST

All included isolates were typed using a-MLVA, with PCR amplification of six variable number of tandem repeats (VNTR) loci, as described by Nielsen et al. [7]. In short, PCR were done as singleplex PCR with unlabelled primers. Subsequent size determination were done using an automated capillary electrophoresis system (QIAxcel, Qiagen) and a high-resolution cartridge [7]. Each of the VNTR loci was manually binned depending on size and assigned a number. As result each isolate were given a six-digit a-MLVA code. For isolates without a measured band size, for a VNTR locus, PCR amplification was performed twice for the given locus. The identified codes were primarily translated to STs by an in-house library, but to further confirm our findings, at least one isolate from each identified a-MLVA code were verified by MLST, with the exception of one a-MLVA code. In groups with several isolates having identical a-MLVA codes, we included isolates with extreme band sizes within bins, to effectively cohere clusters. In the ESBL population we furthermore included isolates, belonging to ST131, for O-serogrouping, performed by GlycoVaxyn. In all, MLST was performed on 31 isolates from the ESBL population, on 45 isolates from the resistant population and on 52 isolates from the susceptible population. MLST was done using the Achtmann MLST scheme with PCR and sequencing of seven housekeeping genes followed by assignment of an allelic number from the MLST-database (http://mlst.warwick.ac.uk/mlst/dbs/Ecoli) [7, 13].

## Results

### Characterization of the *E. coli* populations

**Fig. 1:**
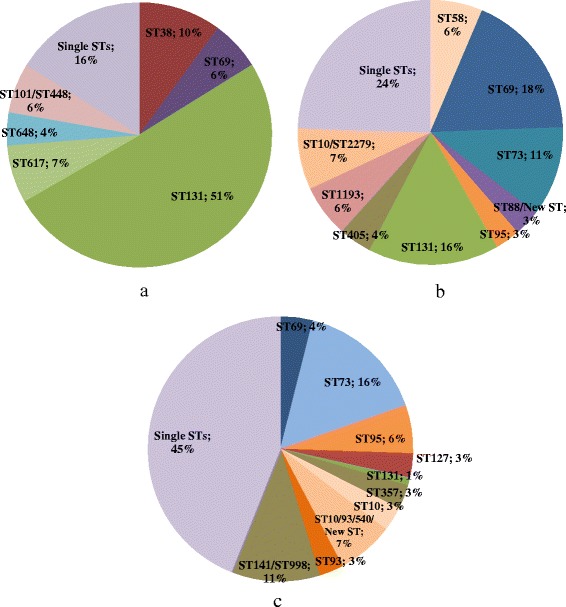
Distribution of major clusters of sequence types among the three *E. coli* populations*.* We show STs making up 2 % or more of isolates—except for ST131 in the susceptible population. Single STs = Percentage of STs found in one isolate only. **a** ESBL-producing *E. coli*. STs found but not shown: ST14, ST62, ST88, ST120, ST224, ST315, ST354, ST428, ST636 and ST2852. There was not found a unique a-MLVA code for ST746/ST1598 and ST101/ST448. **b** Resistant (non-ESBL) *E. coli*. STs found but not shown: ST14, ST38, ST62, ST80, ST135, ST141, ST362, ST372, ST393, ST457, ST648, ST978, ST1597, New ST (1 and 2) and ST117/ST1177. There was not found a unique a-MLVA code for ST10/ST2279, ST88/New ST 3 and ST117/ST1177. **c** Susceptible *E. coli*. STs found but not shown: ST12, ST14, ST38, ST48, ST59, ST62, ST80, ST101, ST127, ST141, ST162, ST405, ST410, ST420, ST501, ST538, ST540, ST582, ST589, ST681, ST714, ST1161, ST1331, ST1444, ST1858, ST3672, ST4235, New ST (4–10) and ST3846. There was not found a unique a-MLVA code for ST10/93/540/New ST and ST141/998

**Fig. 2:**
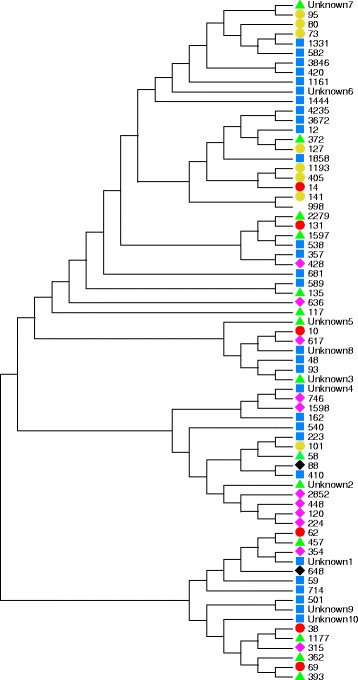
Maximum likelihood tree showing all ST’s found in the three populations. We do not show the number of isolates belonging to each ST.  = ST´s found in the susceptible population only.  = ST´s found in the resistant, non-ESBL population only.  = ST´s found in the ESBL population only.  = ST´s found in the susceptible and resistant, non-ESBL populations.  = ST´s found in all three populations.  = ST´s found in the two resistant populations

We defined a cluster as three or more isolates identified as the same ST or a-MLVA code. a-MLVA was performed on all isolates and MLST was performed to link a-MLVA codes to an international known nomenclature. Used in this way a-MLVA divides isolates before more laborious and costly typing by MLST. A total of 83 unique a-MLVA codes were identified among the 286 isolates (Additional file 1). In several occasions one ST was subdivided into more than one a-MLVA codes and complex situations existed where the a-MLVA method did not distinguish between two or more STs as has been seen previously with this current a-MLVA method as well as with other MLVA methods [7, 14] (Additional file 1). Results from the MLST are presented in Fig. 1a–c and a maximum likelihood tree showing all ST’s found in the three populations is found in Fig. 2. We found 72 different sequence types in the 141 isolates typed by MLST. We discovered ten STs, not previously identified, here labelled “New ST 1–10". We detected a large ST131 cluster with one a-MLVA code primarily belonging to O25, all of which were resistant to ciprofloxacin. A minor ST131 cluster, with another a-MLVA code belonging to O16 was likewise detected, but here no isolates showed resistance to ciprofloxacin.

### Antibiotic resistance patterns

**Fig. 3:**
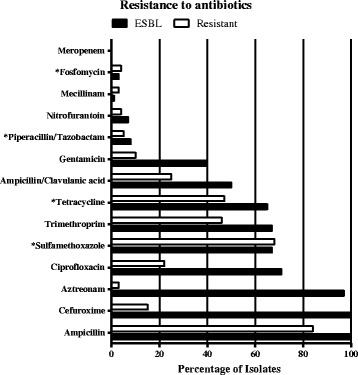
Resistance patterns for the resistant populations

**Fig. 4:**
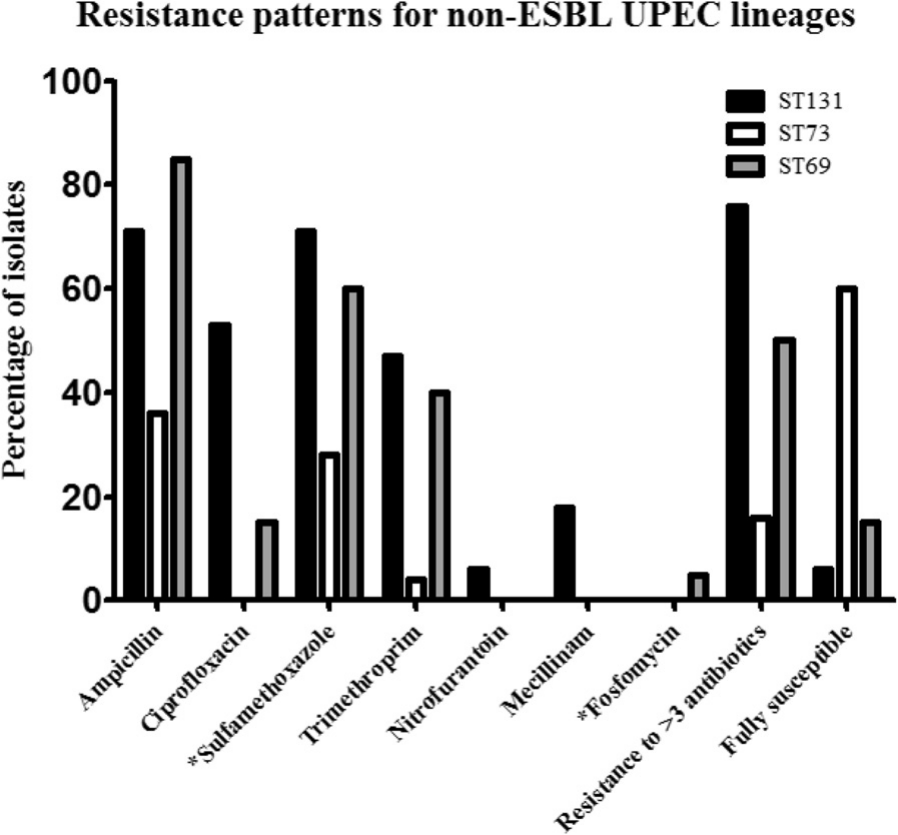
Percentages of isolates resistant to chosen antibiotics used for oral administration. Shown are resistance patterns for three common UPEC ST lineages, where all isolates are non-ESBL-producers but present in either the susceptible or resistant population. N: ST131 = 17, ST73 = 25, ST69 = 20

Results of antibiotic susceptibility testing are found in Figs. 3 and 4. Generally the ESBL population showed high levels of resistance with 100 % of the isolates showing resistance towards three or more of the tested antibiotics. In the resistant population 66 % of the isolates were resistant to ≥3 of the antibiotics. All isolates were susceptible to meropenem and most to mecillinam (97–99 %), fosfomycin (96–97 %), nitrofurantoin (93–96 %), and piperacillin/tazobactam (92–95 %). Figure 3 indicates that resistant (non-ESBL) ST131 have a broader spectrum of resistance than resistant (non-ESBL) ST73 and ST69.

### ESBL-genotyping

We found that 98 % of the ESBL-producing *E. coli* isolates were positive for the presence of a *bla*_CTX-M_. *bla*_CTX-M_ group 1 dominated with 73 % of all isolates carrying this type and 54 % produced CTX-M-15. A total of 24 % of isolates belonged to CTX-M group 9.

## Discussion

This study was carried out to describe the diversity among susceptible, resistant (non-ESBL) and ESBL-producing *E. coli.* MLST was performed to link a-MLVA codes to an international known nomenclature. Overall we found ST131, ST73 and ST69 to be the dominating lineages among all isolates, in accordance with previous studies [15]. The resistant (non-ESBL) ST131 showed a broader spectrum of resistance than the other prevalent STs in this population (ST73 and ST69) (Fig. 3), which has also been previously described by Horner et al. [15]*.* We found that ESBL-producing *E. coli*, resistant *E. coli* and susceptible *E. coli* in turn were dominated by different lineages based on a-MLVA codes and STs (Fig. 1). In general, the susceptible *E. coli* population was a much more diverse group of isolates with more STs and smaller clusters (Fig. 1 and Additional file 1). The ST-clusters in this population were subdivided by a-MLVA, emphasizing a higher level of diversity. The resistant *E. coli* and ESBL-producing *E. coli* were found in larger clusters. Especially the ESBL-population was a more homogenous population with fewest a-MLVA codes and STs (Fig. 1). We found that ST131 completely dominated this ESBL-producing *E. coli* population, as previously seen in the Copenhagen area [7, 16]. Only a single ST131 isolate was found among the susceptible isolates and 14 detected in the resistant population (Fig. 1). Corresponding well with preceding reports, we found that most ST131 isolates belong to serogroup O25 and a minor part being identified as O16 [16, 17]. It should be noted, that the ESBL-population is likely a more selected group, allowing us to detect less successful STs with limited impact as UPEC, which will influence the established representation of diversity in this group. However, we speculate if resistance in different *E. coli* populations are somewhat defined by intrinsic differences in distinct *E. coli* lineages, making a limited number of UPEC ST-lineages capable of obtaining and spreading *bla*_CTX-M_. Some lineages successfully acquire and maintain different types of mobile resistance genes like ST69 and especially ST131, while other *E. coli* lineages remain fairly susceptible and rarely take up plasmids, as seen with ST73 and ST95 [3, 4]. ST131 could be one of the UPEC lineages, among these resistant and ESBL-producing isolates, with the highest ability to colonize the human gut, creating a high prevalence in these populations. Nevertheless, production of ESBLs are found among specific UPEC present, to some extent, in all populations. Thus, antibiotic selection creates a less varied population structure of related isolates while antibiotic free environments allows for competition and diverse non-related population structure [18]. The UPEC we found in our three populations have been found in other population-studies of *E. coli* isolates causing bacteraemia, strongly indicating that *E. coli* isolates capable of one invasive disease can cause severe invasive infections, independent of antibiotic susceptibility [15]. Finally, our results are in line with the reports, using more than one method of characterization, identifying susceptible ST-lineages as more heterogeneous populations [2, 6, 17, 19].

One of the major limitations of this study is the incomplete separation of all STs. However, this also provides high discriminatory power in the ability to separate closely related ST-lineages and thereby describe *E. coli* populations in detail. Yet, the missing identification of STs are unlikely to cause bias in our overall conclusion as the prevalence and identification of the major lineages in the three populations are unique. The a-MLVA typing method was successful in the characterization and sub-division of some large cluster of ST-lineages. The study is additionally limited by the relatively small number of isolates characterized and the limited time period of collection, which does not allow us to identify any fluctuation in dominant lineages of non-ESBL populations.

## Conclusions

In conclusion; this study was carried out to describe the diversity among susceptible, resistant (non-ESBL) and ESBL-producing *E. coli.* Overall we found ST131, ST73 and ST69 to be the dominating lineages among all isolates, in accordance with previous studies [15]. ST131 was able to effectively dominate the ESBL-producing UPEC and seems to be a specialized UPEC adapted as an efficient resistant and ESBL-producing lineage, sustainable in the community once present. The findings we present here strongly suggest that the observed dissemination of ESBL-producing *E. coli* are due to the spread of certain UPEC lineages already present in the general UPEC population [3]. Such lineages seems able to dominate the ESBL-population and it is likely that the spread of resistance occurs due to selection of previously specialized UPEC with limited fitness loss due to ESBL-production [4].

## Availability of supporting data

We have not deposited additional or supporting data online, but we present the data on which our findings are based in the Additional file 1 for the present paper.

## Additional file

Additional file 1: The additional data contains the characterization of the E.coli populations. It shows the distribution of a-MLVA codes and Sequence Types in details in three tables: Additional file 1: S1 to S3. Here we show the number of isolates found in each cluster of STs and a-MLVA codes, respectively. Furthermore, we present each ST and the corresponding a-MLVA code (or a-MLVA codes in case more than one a-MLVA code was identified for a specific ST). In addition, we show cases where more than one ST was identified by the same a-MLVA. Finally, for the ESBL-population, we show the ESBL genotype identified within each a-MLVA code. (PDF 439 101 kb)

## Abbreviations

a-MLVA: abbreviated MLVA
ESBL: extended-spectrum-beta-lactamase
ExPEC: extraintestinal pathogenic *Escherichia coli*
MALDI-TOF: matrix-assisted laser desorption ionization-time of flight mass spectrometry
MLST: multi locus sequence typing
MLVA: multi locus variable number of tandem repeat analysis
PCR: polymerase chain reaction
ST: sequence type
UPEC: uropathogenic *E.coli*
UTI: urinary tract infection
VNTR: variable number of tandem repeats
