# Erratum to: Population structure of Drug-Susceptible,—Resistant and ESBL-producing *Escherichia coli* from community-acquired urinary tract infections

**DOI:** 10.1186/s12866-016-0725-4

**Published:** 2016-06-20

**Authors:** Frederik Boëtius Hertz, Jesper Boye Nielsen, Kristian Schønning, Pia Littauer, Jenny Dahl Knudsen, Anders Løbner-Olesen, Niels Frimodt-Møller

**Affiliations:** Department of Clinical Microbiology, Hvidovre University Hospital, Copenhagen, Denmark; Department of Biology, University of Copenhagen, Copenhagen, Denmark; Depatment of Clinical Microbiology, Rigshospitalet, Copenhagen, Denmark

## Erratum

The original version of this article [1] unfortunately contained a mistake in the title. The original title “Population structure of drugsusceptible,—resistant and ESBL-producing Escherichia coli from community-acquired urinary tract” should be replaced by “Population structure of Drug-Susceptible, -Resistant and ESBL-producing Escherichia coli from Community-Acquired Urinary Tract Infections”.

The original article has been updated to reflect this change.
