# Supplementary material for: “Population structure of drug-susceptible,—resistant and ESBL-producing Escherichia coli from community-acquired urinary tract”
Source: BMC Microbiol. 2016 Apr 11;16:63. doi: 10.1186/s12866-016-0681-z (PMC4827192; doi:10.1186/s12866-016-0681-z)
Supplement: Additional file 1: — The additional data contains the characterization of the E.coli populations. It shows the distribution of a-MLVA codes and Sequence Types in details in three tables: Additional file 1: S1 to S3. Here we show the number of isolates found in each cluster of STs and a-MLVA codes, respectively. Furthermore, we present each ST and the corresponding a-MLVA code (or a-MLVA codes in case more than one a-MLVA code was identified for a specific ST). In addition, we show cases where more than one ST was identified by the same a-MLVA. Finally, for the ESBL-population, we show the ESBL genotype identified within each a-MLVA code. (PDF 439 101 kb) [file 12866_2016_681_MOESM1_ESM.pdf]

Characterization of *E.coli* populations

Distribution of a-MLVA codes and Sequence Types in Supplementary materials 1-3

Distributions of a-MLVA codes and sequence types in the three *E. coli* populations are found in detail in Supplementary materials 1-3. We show:

1) The number of isolates found in each cluster of sequence types and a-MLVA codes
2) The sequence type and the corresponding a-MLVA code (or a-MLVA codes in case more than one a-MLVA code was identified for the specific ST)
3) We also show cases where more than one ST was identified by the same a-MLVA (shown as ST10/ST617, for example)
4) Finally, for the ESBL-population, we show the ESBL genotype identified within the a-MLVA code

Abbreviated-MLVA codes

PCR amplification of the six MLVA alleles was done as described in the primary manuscript and this abbreviated MLVA (a-MLVA) was used to type the three strain collections. As such, all isolates (100%) were typable by this a-MLVA method. All but one isolate, selected for MLST, had the alleles successfully sequenced. However, a single isolate with a unique a-MLVA code was not sent for MLST.

In these three populations we found a total of 83 a-MLVA-codes and 72 sequence types.

Thirteen a-MLVA´s and 13 ST´s were found in two out of three populations. Six a-MLVA´s and seven ST´s were found in all three populations.

Ten a-MLVA codes were unique and classified as “unknown” sequence types (in Supplementary material classified as “New STs”).

Overall, ST131 constituted 23% of all isolates and ST69 constituted 10%. ST73 was found to constitute 9%. Of the found a-MLVA codes, 74 identified just one sequence type. Yet, in quite a few situations one specific ST was subdivided by more than one a-MLVA code. More complex situations were seen where several different ST´s were assigned the same a-MLVA code.

As result:

- ST 10 were subdivided by three different a-MLVA codes
- ST38 by three codes
- ST69 by three codes
- ST73 by ten codes
- ST95 by three codes
- ST141 by three codes
- ST357 by two codes
- ST405 by two codes
- ST648 by two codes and lastly
- ST131 were subdivided by four different codes.

Equally, the a-MLVA method could not distinguish certain sequence types and in particular eight a-MLVA-codes did not classify unique sequence types:

- ST58, ST101 and ST448 shared one a-MLVA code. ST58 and ST448 are double-locus variants.
- ST998 and one of the a-MLVA codes for ST141 were identical, were ST141 and ST998 are single-locus variants.
- It was not possible to discriminate between on isolate of ST14 and ST1193 which are also single-locus variants.
- ST38, ST117 and ST1177 were assigned a single a-MLVA code. Here ST38 and ST1177 are single locus variant with ST117 being unrelated.
- One a-MLVA code for ST10 also identified ST746, ST1598 and one New ST. Here ST10 and ST746 are single-locus variants while the new ST is a double-locus variant of ST10.
- A different ST10 a-MLVA code was identical to codes for ST93, ST540, ST617, ST2279 and a New ST where only ST617 belonged to ST Complex 10 and was a double-locus variant of ST10.
- Also ST354 and a New ST had the same code.
- Finally ST88, ST410 and one New ST had a common code with ST88 and ST410 being double locus variants and belonging to ST Complex 23.

Supplementary material 1. Characterization for ESBL-producing *E. coli.* ST131 was identified by three a-MLVA codes. ST101 and ST448 shared one a-MLVA code. ST746 and ST1598 shared one a-MLVA code. CTX-M-79 and CTX-M-55 could not be separated.

Number of isolates found in cluster	Sequence Types identified within the a-MLVA code	a-MLVA code	ESBL Genotype identified within the a-MLVA code
1	ST14	123645	TEM-1
10	ST38	173050	CTX-M-14
1	ST62	161370	CTX-M-14
6	ST69	173277	CTX-M-14/27/(79/55)/TEM-1
1	ST88	132261	CTX-M-1
1	ST120	132271	CTX-M-15
1	ST131	103562	CTX-M-14
44	ST131	153562	CTX-M-14/15/27/28/(79/55)
5	ST131	163562	CTX-M-15/CTX-M-1
1	ST224	131271	CTX-M-15
1	ST315	173552	CTX-M-14
1	ST354	151170	CTX-M-14
1	ST428	252365	CTX-M-1
7	ST10/ST617	132251	CTX-M-15/28/(79/55)
1	ST636	293852	CTX-M-15
4	ST648	161160	CTX-M-15
2	ST998	224665	CTX-M-1/15
2	ST2852	131291	CTX-M-15
6	ST101/ST448	131261	CTX-M-1/15/(79/55)
2	ST746/ST1598	131251	CTX-M-15/28

Supplementary material 2. Characterization of resistant *E.coli* (non-ESBL). ST10, ST69, ST73, ST95, ST131 and ST405 were identified by different a-MLVA codes. ST10 and ST2279 shared one a-MLVA code. ST117 and ST1177 shared one a-MLVA code.

Number of isolates found in cluster	Sequence Types identified within the a-MLVA code	a-MLVA
2	ST10	124643
7	ST10/ST2279	132251
1	ST14	123645
1	ST38	173051
6	ST58	131261
1	ST62	161370
2	ST69	173270
15	ST69	173277
1	ST73	176655
1	ST73	266655
8	ST73	276655
2	ST80	254575
3	ST88/New ST	132261
1	ST95	223643
2	ST95	223653
1	ST127	264953
1	ST131	143562
10	ST131	153562
3	ST131	163562
1	ST135	225661
1	ST141	124665
1	ST362	143250

1	ST372	224563
1	ST393	171577
2	ST405	121250
2	ST405	131250
1	ST457	161371
1	ST648	161150
1	ST978	226565
6	ST1193	124645
2	ST1597	162562
2	ST117/ST1177	173050
1	New ST	123552
1	New ST	131251
1	New ST	162438
1	MLST NOT PERFORMED	266561

Supplementary material 3. Characterization of susceptible *E.coli.* ST10, ST59, ST69, ST73, ST95, ST127 and ST357 were each identified by different a-MLVA codes. ST10/ST93/ST540/New ST shared one a-MLVA code. ST141 and ST998 shared one a-MLVA code. ST420 and ST3846 shared one a-MLVA code.

Number of isolates found in cluster	Sequence Types identified within the a-MLVA code	a-MLVA
2	ST12	266562
2	ST14	124645
2	ST38	253263
2	ST48	131281
1	ST59	241370
1	ST59	251380
1	ST62	161370
1	ST69	103277
3	ST69	173277
1	ST73	176655
1	ST73	203655
1	ST73	206653
2	ST73	206655
1	ST73	256653
2	ST73	266655
1	ST73	276555
1	ST73	276655
4	ST73	276655
1	ST73	276665
1	ST80	254575
1	ST95	203653
5	ST95	223653
2	ST101	131261

2	ST127	264953
1	ST127	274953
1	ST131	153562
2	ST141	204665
1	ST162	211251
3	ST223	101261
1	ST357	150365
2	ST357	152365
1	ST405	131250
1	ST410	132261
1	ST501	163777
1	ST538	152363
1	ST582	274242
1	ST589	287572
1	ST681	224452
1	ST714	473050
1	ST1161	124675
1	ST1331	274655
1	ST1444	273673
1	ST1858	224645
1	ST3672	226962
2	ST4235	224743
1	New ST	151170
1	New ST	112871

1	New ST	272275
1	New ST	232251
1	New ST	171250
3	ST10	131251
7	ST10/ST93/ST540/New ST	132251
10	ST141/ST998	224665
2	ST420/ST3846	254665
